# 4549 Reproducible Informatics for Reproducible Translational Research

**DOI:** 10.1017/cts.2020.221

**Published:** 2020-07-29

**Authors:** Ram Gouripeddi, Katherine Sward, Mollie Cummins, Karen Eilbeck, Bernie LaSalle, Julio C. Facelli

**Affiliations:** 1 University of Utah School of Medicine; 2 University of Utah

## Abstract

OBJECTIVES/GOALS: Characterize formal informatics methods and approaches for enabling reproducible translational research. Education of reproducible methods to translational researchers and informaticians. METHODS/STUDY POPULATION: We performed a scoping review [1] of selected informatics literature (e.g. [2,3]) from PubMed and Scopus. In addition we reviewed literature and documentation of translational research informatics projects [4–21] at the University of Utah. RESULTS/ANTICIPATED RESULTS: The example informatics projects we identified in our literature covered a broad spectrum of translational research. These include research recruitment, research data requisition, study design and statistical analysis, biomedical vocabularies and metadata for data integration, data provenance and quality, and uncertainty. Elements impacting reproducibility of research include (1) Research Data: its semantics, quality, metadata and provenance; and (2) Research Processes: study conduct including activities and interventions undertaken, collections of biospecimens and data, and data integration. The informatics methods and approaches we identified as enablers of reproducibility include the use of templates, management of workflows and processes, scalable methods for managing data, metadata and semantics, appropriate software architectures and containerization, convergence methods and uncertainty quantification. In addition these methods need to be open and shareable and should be quantifiable to measure their ability to achieve reproducibility. DISCUSSION/SIGNIFICANCE OF IMPACT: The ability to collect large volumes of data collection has ballooned in nearly every area of science, while the ability to capturing research processes hasn’t kept with this pace. Potential for problematic research practices and irreproducible results are concerns.

Reproducibility is a core essentially of translational research. Translational research informatics provides methods and means for enabling reproducibility and FAIRness [22] in translational research. In addition there is a need for translational informatics itself to be reproducible to make research reproducible so that methods developed for one study or biomedical domain can be applied elsewhere. Such informatics research and development requires a mindset for meta-research [23].

The informatics methods we identified covers the spectrum of reproducibility (computational, empirical and statistical) and across different levels of reproducibility (reviewable, replicable, confirmable, auditable, and open or complete) [24–29]. While there are existing and ongoing efforts in developing informatics methods for translational research reproducibility in Utah and elsewhere, there is a need to further develop formal informatics methods and approaches: the Informatics of Research Reproducibility.

In this presentation, we summarize the studies and literature we identified and discuss our key findings and gaps in informatics methods for research reproducibility. We conclude by discussing how we are covering these topics in a translational research informatics course.

1. Pham MT, Rajić A, Greig JD, Sargeant JM, Papadopoulos A, McEwen SA. A scoping review of scoping reviews: advancing the approach and enhancing the consistency. Res Synth Methods. 2014 Dec;5(4):371–85.
2. McIntosh LD, Juehne A, Vitale CRH, Liu X, Alcoser R, Lukas JC, Evanoff B. Repeat: a framework to assess empirical reproducibility in biomedical research. BMC Med Res Methodol [Internet]. 2017 Sep 18 [cited 2018 Nov 30];17. Available from: https://www.ncbi.nlm.nih.gov/pmc/articles/PMC5604503/
3. Denaxas S, Direk K, Gonzalez-Izquierdo A, Pikoula M, Cakiroglu A, Moore J, Hemingway H, Smeeth L. Methods for enhancing the reproducibility of biomedical research findings using electronic health records. BioData Min. 2017;10:31.
4. Burnett N, Gouripeddi R, Wen J, Mo P, Madsen R, Butcher R, Sward K, Facelli JC. Harmonization of Sensor Metadata and Measurements to Support Exposomic Research. In: 2016 International Society of Exposure Science [Internet]. Research Triangle Park, NC, USA; 2017 [cited 2017 Jun 17]. Available from: http://www.intlexposurescience.org/ISES2017
5. Butcher R, Gouripeddi RK, Madsen R, Mo P, LaSalle B. CCTS Biomedical Informatics Core Research Data Service. In Salt Lake City; 2016.
6. Cummins M, Gouripeddi R, Facelli J. A low-cost, low-barrier clinical trials registry to support effective recruitment. In Salt Lake City, Utah, USA; 2016 [cited 2018 Nov 30]. Available from: //campusguides.lib.utah.edu/UtahRR16/abstracts
7. Gouripeddi R, Warner P, Madsen R, Mo P, Burnett N, Wen J, Lund A, Butcher R, Cummins MR, Facelli J, Sward K. An Infrastructure for Reproducibile Exposomic Research. In: Research Reproducibility 2016 [Internet]. Salt Lake City, Utah, USA; 2016 [cited 2018 Nov 30]. Available from: //campusguides.lib.utah.edu/UtahRR16/abstracts
8. Eilbeck K, Lewis SE, Mungall CJ, Yandell M, Stein L, Durbin R, Ashburner M. The Sequence Ontology: a tool for the unification of genome annotations. Genome Biol. 2005;6:R44.
9. Gouripeddi R, Cummins M, Madsen R, LaSalle B, Redd AM, Presson AP, Ye X, Facelli JC, Green T, Harper S. Streamlining study design and statistical analysis for quality improvement and research reproducibility. J Clin Transl Sci. 2017 Sep;1(S1):18–9.
10. Gouripeddi R, Eilbeck K, Cummins M, Sward K, LaSalle B, Peterson K, Madsen R, Warner P, Dere W, Facelli JC. A Conceptual Architecture for Reproducible On-demand Data Integration for Complex Diseases. In: Research Reproducibility 2016 (UtahRR16) [Internet]. Salt Lake City, Utah, USA; 2016 [cited 2017 Apr 25]. Available from: https://zenodo.org/record/168067
11. Gouripeddi R, Lane E, Madsen R, Butcher R, LaSalle B, Sward K, Fritz J, Facelli JC, Cummins M, Shao J, Singleton R. Towards a scalable informatics platform for enhancing accrual into clinical research studies. J Clin Transl Sci. 2017 Sep;1(S1):20–20.
12. Gouripeddi R, Deka R, Reese T, Butcher R, Martin B, Talbert J, LaSalle B, Facelli J, Brixner D. Reproducibility of Electronic Health Record Research Data Requests. In Washington, DC, USA; 2018 [cited 2018 Apr 21]. Available from: https://zenodo.org/record/1226602#.WtvvyZch270
13. Gouripeddi R, Mo P, Madsen R, Warner P, Butcher R, Wen J, Shao J, Burnett N, Rajan NS, LaSalle B, Facelli JC. A Framework for Metadata Management and Automated Discovery for Heterogeneous Data Integration. In: 2016 BD2K All Hands Meeting [Internet]. Bethesda, MD; November 29-30 [cited 2017 Apr 25]. Available from: https://zenodo.org/record/167885
14. Groat D, Gouripeddi R, Lin YK, Dere W, Murray M, Madsen R, Gestaland P, Facelli J. Identification of High-Level Formalisms that Support Translational Research Reproducibility. In: Research Reproducibility 2018 [Internet]. Salt Lake City, Utah, USA; 2018 [cited 2018 Oct 30]. Available from: //campusguides.lib.utah.edu/UtahRR18/abstracts
15. Huser V, Kahn MG, Brown JS, Gouripeddi R. Methods for examining data quality in healthcare integrated data repositories. Pac Symp Biocomput Pac Symp Biocomput. 2018;23:628–33.
16. Lund A, Gouripeddi R, Burnett N, Tran L-T, Mo P, Madsen R, Cummins M, Sward K, Facelli J. Enabling Reproducible Computational Modeling: The Utah PRISMS Ecosystem. In Salt Lake City, Utah, USA; 2018 [cited 2018 Oct 30]. Available from: //campusguides.lib.utah.edu/UtahRR18/abstracts
17. Pflieger LT, Mason CC, Facelli JC. Uncertainty quantification in breast cancer risk prediction models using self-reported family health history. J Clin Transl Sci. 2017 Feb;1(1):53–9.
18. Shao J, Gouripeddi R, Facelli J. Improving Clinical Trial Research Reproducibility using Reproducible Informatics Methods. In Salt Lake City, Utah, USA; 2018 [cited 2018 Oct 30]. Available from: //campusguides.lib.utah.edu/UtahRR18/abstracts
19. Shao J, Gouripeddi R, Facelli JC. Semantic characterization of clinical trial descriptions from ClincalTrials.gov and patient notes from MIMIC-III. J Clin Transl Sci. 2017 Sep;1(S1):12–12.
20. Tiase V, Gouripeddi R, Burnett N, Butcher R, Mo P, Cummins M, Sward K. Advancing Study Metadata Models to Support an Exposomic Informatics Infrastructure. In Ottawa, Canada; 2018 [cited 2018 Oct 30]. Available from: = http://www.eiseverywhere.com/ehome/294696/638649/?&t=8c531cecd4bb0a5efc6a0045f5bec0c3
21. Wen J, Gouripeddi R, Facelli JC. Metadata Discovery of Heterogeneous Biomedical Datasets Using Token-Based Features. In: IT Convergence and Security 2017 [Internet]. Springer, Singapore; 2017 [cited 2017 Sep 6]. p. 60–7. (Lecture Notes in Electrical Engineering). Available from: https://link.springer.com/chapter/10.1007/978-981-10-6451-7_8
22. Wilkinson MD, Dumontier M, Aalbersberg IjJ, Appleton G, Axton M, Baak A, Blomberg N, Boiten J-W, da Silva Santos LB, Bourne PE, Bouwman J, Brookes AJ, Clark T, Crosas M, Dillo I, Dumon O, Edmunds S, Evelo CT, Finkers R, Gonzalez-Beltran A, Gray AJG, Groth P, Goble C, Grethe JS, Heringa J, ’t Hoen PAC, Hooft R, Kuhn T, Kok R, Kok J, Lusher SJ, Martone ME, Mons A, Packer AL, Persson B, Rocca-Serra P, Roos M, van Schaik R, Sansone S-A, Schultes E, Sengstag T, Slater T, Strawn G, Swertz MA, Thompson M, van der Lei J, van Mulligen E, Velterop J, Waagmeester A, Wittenburg P, Wolstencroft K, Zhao J, Mons B. The FAIR Guiding Principles for scientific data management and stewardship. Sci Data. 2016 Mar 15;3:160018.
23. Ioannidis JPA. Meta-research: Why research on research matters. PLOS Biol. 2018 Mar 13;16(3):e2005468.
24. Stodden V, Borwein J, Bailey DH. Setting the default to reproducible. Comput Sci Res SIAM News. 2013;46(5):4–6.
25. Stodden V, McNutt M, Bailey DH, Deelman E, Gil Y, Hanson B, Heroux MA, Ioannidis JPA, Taufer M. Enhancing reproducibility for computational methods. Science. 2016 Dec 9;354(6317):1240–1.
26. Stodden V, McNutt M, Bailey DH, Deelman E, Gil Y, Hanson B, Heroux MA, Ioannidis JPA, Taufer M. Enhancing reproducibility for computational methods. Science. 2016 Dec 9;354(6317):1240–1.
27. Stodden V. Reproducible Research for Scientific Computing: Tools and Strategies for Changing the Culture. Comput Sci Eng. 2012 Jul 1;14(4):13–7.
28. Baker M. Muddled meanings hamper efforts to fix reproducibility crisis. Nat News Available from: http://www.nature.com/news/muddled-meanings-hamper-efforts-to-fix-reproducibility-crisis-1.20076
29. Barba LA. Terminologies for Reproducible Research. ArXiv180203311 Cs 2018 Feb 9; Available from: http://arxiv.org/abs/1802.03311

